# Evaluation of the Risk Prediction Model for Frailty in Diabetic Patients: A Systematic Review and Meta‐Analysis

**DOI:** 10.1155/jdr/4938173

**Published:** 2026-07-09

**Authors:** Qing Chen, Meiling Yang, Mengmeng Chen, Chuyuan Miao, Zidan Wang, Joanne Wai Yee Chung

**Affiliations:** ^1^ School of Nursing, Guangzhou Medical University, Guangzhou, Guangdong, China, gzhmc.edu.cn; ^2^ Department of Nursing, Shenzhen Nanshan People′s Hospital, Shenzhen, Guangdong, China; ^3^ School of Nursing, Capital Medical University, Beijing, China, ccmu.edu.cn; ^4^ Kiang Wu Nursing College of Macau, Macau, China, kwnc.edu.mo

**Keywords:** diabetes mellitus, frailty, prediction models, systematic review

## Abstract

**Background:**

Frailty is highly prevalent among patients with diabetes and is associated with an increased risk of disability, hospitalization, and mortality. Although several frailty prediction models have been developed for this population, their methodological quality, predictive performance, and clinical applicability remain unclear. This systematic review and meta‐analysis was therefore conducted to comprehensively evaluate existing prediction models for frailty in patients with diabetes.

**Methods:**

PubMed, Web of Science, Embase, Cochrane Library, China National Knowledge Infrastructure (CNKI), Wanfang Data, China Biology Medicine Database (CBM), and China Science and Technology Journal Database (VIP) were searched for eligible prediction model studies from database inception to April 28, 2026. Two reviewers independently screened studies, extracted data, and assessed methodological quality using the CHARMS checklist and the PROBAST tool. Random‐effects or fixed‐effect models were applied according to heterogeneity. Meta‐analyses of pooled model discrimination (area under the curve [AUC]) and common predictors were performed using RevMan 5.4 and MedCalc 23.6.1 software.

**Results:**

Of the 3492 identified articles, 19 studies were included, comprising a total of 36 prediction models. Sample sizes ranged from 152 to 1436 participants, and the AUC values varied from 0.703 to 0.975. The random forest model demonstrated the highest discriminative performance (AUC = 0.975). Frequently identified predictors included age, depression, activities of daily living (ADL), nutritional status, duration of diabetes, physical activity, polypharmacy, glycated hemoglobin (HbA1c), cognitive function, and marital status. All studies were judged to have a high risk of bias due to insufficient reporting of participants, predictors, outcomes, and analytical methods, although their overall applicability was considered high.

**Conclusion:**

Existing frailty prediction models for patients with diabetes demonstrated good overall predictive performance and potential clinical utility. Nevertheless, substantial methodological limitations and a high risk of bias were identified across all included studies. Future model development should emphasize methodological rigor, external validation, and transparent reporting to improve reliability and facilitate clinical implementation.

## 1. Introduction

Diabetes mellitus (DM) is a chronic metabolic disorder characterized by persistent hyperglycemia resulting from defects in insulin secretion, insulin action, or both [1]. Due to population aging and lifestyle changes, the prevalence of diabetes continues to rise worldwide. In 2021, an estimated 537 million adults were living with diabetes globally, and this number is projected to reach 783 million by 2045 [2]. China carries the largest diabetes burden globally, with the number of individuals affected expected to increase from 141 million in 2021 to 164 million by 2030 and 175 million by 2045 [3]. Furthermore, according to the International Diabetes Federation, China had the largest population of older adults with diabetes (aged ≥ 65 years) globally, totaling approximately 35.5 million in 2019 [4].

In recent years, frailty has been recognized as the third major complication of diabetes, following macrovascular and microvascular diseases [5]. Frailty is an age‐related syndrome in the elderly, characterized by weight loss, weakened grip strength, slowed walking speed, reduced physical activity, and fatigue. It reflects a decline in physiological reserves, multisystem dysfunction, and increased susceptibility to stress [6, 7]. It predominantly occurs in older adults with comorbidities [8]. Studies have shown that long‐term hyperglycemia, insulin resistance, and oxidative stress in patients with diabetes may increase the risk of frailty [9]. The prevalence of frailty among individuals with diabetes can be as high as 61.8%, which is three times higher than in nondiabetic individuals [10].

This syndrome may not only lead to decreased mobility and greater difficulty in blood glucose monitoring and management among diabetic patients, but also increase the risk of adverse clinical events such as hypoglycemia, falls, infections, delirium, hospitalization, delayed recovery, and death [11]. Although nonpharmacological management, such as physical exercise and healthy diet, along with antidiabetic medications, has been applied in the treatment of diabetic patients with frailty [12], the effects are limited. Therefore, early identification and prevention are crucial components in the care and treatment of diabetic frailty.

The risk prediction model can identify high‐risk individuals, thereby enabling precise prevention, enhancing the quality and efficiency of healthcare [13]. Recently, frailty has emerged as a significant research focus, with increasing attention on developing predictive models for frailty risk in diabetic patients. Despite the development of numerous analytical prediction models, the quality of their methods, predictive performance, and clinical applicability remain unclear. There is no consensus yet on the most effective model for predicting the risk of diabetic frailty, as its occurrence and progression result from the combined effects of multiple predictive factors [14]. Current guidelines primarily assess the risk of frailty in diabetes based on the frailty mechanisms and diabetes characteristics [11], potentially overlooking other important coexisting predictors. Thus, identifying these additional risk predictors is essential for more effectively preventing frailty in diabetic patients.

Therefore, this study is aimed at systematically reviewing and meta‐analyzing existing prediction models for frailty in patients with diabetes. By synthesizing the available evidence, we aim to identify the most frequently reported predictors of frailty, evaluate the predictive performance and methodological quality of existing models, and assess their risk of bias and applicability. The findings of this review may provide an evidence base for future model development, validation, and clinical implementation, thereby facilitating the early identification of patients at high risk of frailty and supporting the development of targeted prevention and management strategies. To ensure conceptual consistency, this review focused on prediction models for frailty assessed using validated frailty instruments. Studies evaluating specific frailty subtypes, including cognitive frailty, oral frailty, psychological frailty, and social frailty, were excluded because these constructs differ from frailty in their underlying mechanisms, assessment approaches, and clinical implications [15].

## 2. Materials and Methods

This systematic review and meta‐analysis was conducted and reported in accordance with the Preferred Reporting Items for Systematic Reviews and Meta‐Analyses (PRISMA) guidelines. The review protocol was prospectively registered in PROSPERO (CRD42024629456).

### 2.1. Problem Formulation

The evidence‐based question was constructed based on the PICOTS framework recommended by the Cochrane Methodology Group [16]. The target population (P) comprised patients with DM. The index prediction models (I) were models developed to predict the risk of frailty in patients with diabetes. The comparator (C) was no competing model. The outcome (O) was the occurrence of frailty among patients with diabetes. The timing of model use (T) was to predict the risk of future frailty by assessing variables such as basic demographic characteristics, lifestyle information, comorbidities, and laboratory test results of diabetic patients. The model usage setting (S) is a hospital clinical environment or a community medical center.

### 2.2. Search Strategy

**Figure 1:**
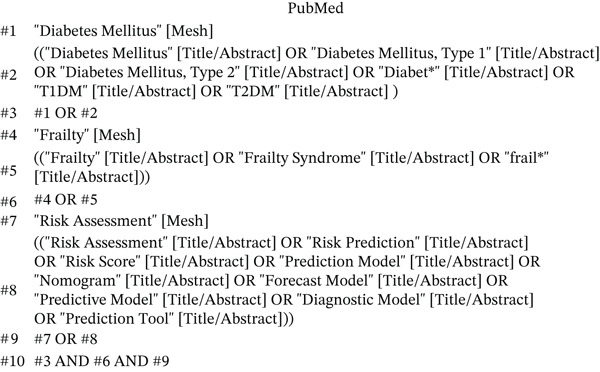
PubMed search strategy.

A comprehensive literature search was conducted to identify studies on predictive models for frailty risk in patients with diabetes, published in both Chinese and English. The search was conducted across seven databases: China National Knowledge Infrastructure (CNKI), Wanfang Data Knowledge Service Platform, China Biology Medicine Database (CBM), China Science and Technology Journal Database (VIP), PubMed, Web of Science, Cochrane Library, and Embase. All records from database inception to April 28, 2026, were considered. A combination of subject headings (e.g., MeSH in PubMed) and free‐text keywords was used. Relevant search terms were categorized as follows: for diabetes, “Diabetes mellitus,” “Diabet ^∗^,” “Diabetes Mellitus, Type 1,” “Diabetes Mellitus, Type 2,” “T1DM,” “T2DM”; for frailty, “Frailty,” “Frailty Syndrome,” “frail ^∗^”; and for predictive models, “Risk Assessment,” “Risk Prediction,” “Risk Score,” “Prediction Model,” “Prediction Tool,” “Forecast Model,” “Nomogram,” “Predictive Model,” and “Diagnostic Model.” Boolean operators (AND and OR) were applied to combine search terms appropriately. For example, the search strategy used in PubMed is shown in Figure 1.

### 2.3. Inclusion and Exclusion Criteria for Literature

The inclusion criteria were as follows: (1) individuals diagnosed with diabetes according to established diagnostic guidelines or expert consensus, irrespective of comorbidities (e.g., hypertension or chronic obstructive pulmonary disease); (2) studies that developed and/or validated prediction models for assessing the risk of frailty in patients with diabetes; (3) cohort, case–control, or cross‐sectional studies; and (4) articles published in either English or Chinese.

The exclusion criteria were as follows: (1) studies that only explored risk factors without developing or validating a prediction model; (2) studies focusing exclusively on specific frailty domains, such as cognitive frailty, oral frailty, psychological frailty, or social frailty; (3) duplicate publications; (4) studies with incomplete data or unavailable full texts; and (5) case reports, reviews, systematic reviews, meta‐analyses, conference abstracts, or other nonoriginal research articles.

### 2.4. Study Selection and Data Extraction

Two reviewers independently screened the retrieved studies according to the predefined eligibility criteria. EndNote 21 software was used for literature management and duplicate removal. Any disagreements during the screening process were resolved through discussion, and a third reviewer was consulted when consensus could not be reached. Standardized data extraction forms were developed based on the Critical Appraisal and Data Extraction for Systematic Reviews of Prediction Modelling Studies (CHARMS) checklist [17]. The extracted data included the first author, publication year, country, study design, study population, outcome measures, modeling methods, predictor selection strategies, candidate predictors, sample size, handling of missing data, number of predictors, model performance, validation methods, and model presentation format.

### 2.5. Quality Assessment

Two reviewers independently assessed the risk of bias and applicability of the included studies using the Prediction Model Risk of Bias Assessment Tool (PROBAST) [18]. Risk of bias was evaluated across four domains: participants, predictors, outcome, and analysis. Applicability was assessed across three domains: participants, predictors, and outcome. Any disagreements were resolved through discussion, with consultation from a third reviewer when necessary.

### 2.6. Statistical Analysis Methods

Meta‐analyses were performed using RevMan 5.4 and MedCalc Version 23.6.1 software. MedCalc was used to pool the area under the receiver operating characteristic curve (AUC) values reported by the included prediction models. Standard errors were calculated from the reported AUC values and their corresponding 95% confidence intervals (CIs). Heterogeneity among studies was assessed using Cochran′s Q test and the *I* ^2^ statistic. A fixed‐effect model was applied when heterogeneity was low (*I* ^2^ < 50% and *p* > 0.10); otherwise, a random‐effects model was used (*I* ^2^ ≥ 50% or *p* ≤ 0.10). The pooled effect estimate for model performance was expressed as the pooled AUC with corresponding 95% CIs.

In addition, meta‐analyses of predictors were conducted using RevMan 5.4. Predictors reported in at least two studies with comparable definitions were quantitatively synthesized. Odds ratios (ORs) and corresponding 95% CIs were calculated as summary effect estimates. Heterogeneity was assessed using Cochran′s Q test and the *I* ^2^ statistic, and fixed‐effect or random‐effects models were applied as appropriate. Statistical significance was defined as *p* < 0.05.

## 3. Results

### 3.1. Study Selection

**Figure 2:**
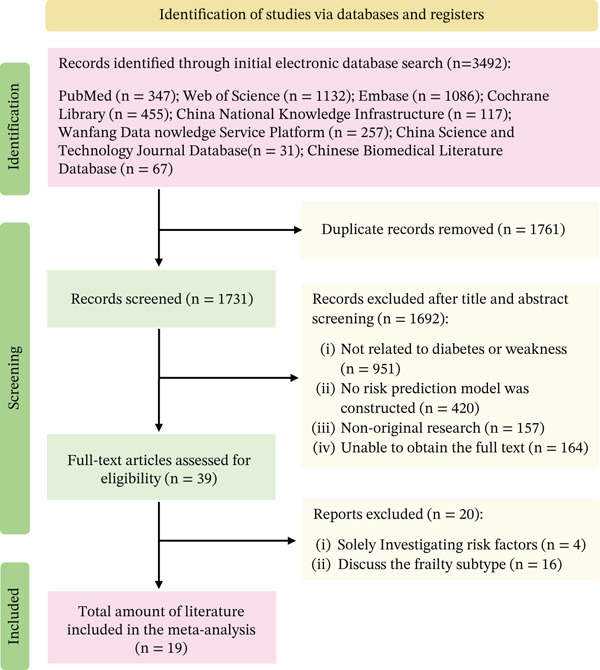
Flowchart of the study selection.

Figure 2 presents the study selection process. A total of 3492 records were identified through database searching, of which 1761 duplicate records were removed. After screening titles and abstracts, 1731 records were excluded. The remaining 39 articles underwent full‐text assessment for eligibility, and 19 studies [19–37] were ultimately included in the review.

### 3.2. Basic Characteristics of the Included Literature

Among the 19 included studies [19–37], all were published between 2023 and 2026. Seventeen studies were published in Chinese [21–37], whereas two studies were published in English [19, 20]. Regarding study design, 17 studies were cross‐sectional studies [20–34, 36, 37], and two studies [19, 35] were retrospective cohort studies, both of which were based on the China Health and Retirement Longitudinal Study (CHARLS) database. All studies were conducted in China. Three studies [30, 33, 37] were multicenter studies involving two centers, whereas the remaining studies were conducted at a single center.

Frailty was assessed using four validated instruments. The FRAIL Scale was used in seven studies [22, 23, 25, 26, 29, 31, 34], the Fried Frailty Phenotype (FP) in eight studies [19, 20, 24, 28, 30, 33, 35, 37], the Tilburg Frailty Indicator (TFI) in three studies [21, 27, 36], and the Edmonton Frail Scale (EFS) in one study [32]. Among these instruments, the FRAIL Scale and the FP were the most frequently used. The prevalence of frailty among patients with diabetes ranged from 10.1% to 51.2%, with a median prevalence of 37.1%. The highest prevalence was reported by Yin YuanYuan [21] (51.2%), whereas the lowest prevalence was reported by Bu et al. [19] (10.1%). The basic characteristics of the included studies are presented in Table 1.

**Table 1:** Basic characteristics of the included studies (*n* = 19).

Included studies	Time	Country	Study design	Participants	Data source	Outcome measure	Incidence of frailty
Bu F et al. [19]	2023	China	Retrospective study	Patients with diabetes (no clear age limit, actually included < 55 years)	CHARLS	FP	10.1%
Yin YuanYuan[21]	2024	China	Cross‐sectional study	Elderly inpatients with diabetes (≥ 60 years)	A single‐center tertiary hospital	TFI	51.2%
Dang Xue [22]	2024	China	Cross‐sectional study	Elderly inpatients with diabetes (≥65 years)	A single‐center tertiary hospital	FRAIL	32.0%
Liu Xi et al. [23]	2024	China	Cross‐sectional study	Elderly inpatients with diabetes (≥ 60 years)	A single‐center tertiary hospital	FRAIL	28.4%
Tang Qingfeng et al. [24]	2024	China	Cross‐sectional study	Elderly inpatients with diabetes (≥ 60 years)	A single‐center tertiary hospital	FP	37.6%
Xi Mengxing [25]	2024	China	Cross‐sectional study	Elderly inpatients with diabetes (≥ 60years)	A single‐center tertiary hospital	FRAIL	38.5%
Li Teng [26]	2024	China	Cross‐sectional study	Elderly inpatients with diabetes (≥ 60years)	A single‐center tertiary hospital	FRAIL	44.3%
Du Jin et al. [20]	2024	China	Cross‐sectional study	Community‐dwelling elderly with type 2 diabetes (≥65 years)	Community hospital	FP	18.1%
Wang Bingjie et al. [27]	2024	China	Cross‐sectional study	Elderly inpatients with diabetic foot (≥ 60 years)	A dual‐center tertiary hospital	TFI	44.0%
Wang Zhuo et al. [28]	2025	China	Cross‐sectional study	Elderly patients with type 2 diabetes (≥60 years)	A single‐center tertiary hospital	FP	31.1%
Dong Xuting et al. [29]	2023	China	Cross‐sectional study	Elderly patients with diabetic retinopathy (≥ 60 years)	A single‐center tertiary hospital	FRAIL	43.5%
Zheng Xuemei et al. [30]	2024	China	Cross‐sectional study	Elderly inpatients with diabetes (≥ 60 years)	A dual‐center tertiary hospital	FP	29.5%
Chen Youmei [31]	2024	China	Cross‐sectional study	Elderly inpatients with diabetes (≥ 60 years)	A single‐center tertiary hospital	FRAIL	37.2%
Deng Shunzhi et al. [36]	2024	China	Cross‐sectional study	Elderly inpatients with diabetes(≥ 65years)	A single‐center tertiary hospital	TFI	43.6%
Ma Bingying [37]	2024	China	Cross‐sectional study	Elderly patients with type 2 diabetes(≥ 60years)	A single‐center tertiary hospital	FP	31.5%
Wang Weijia [34]	2024	China	Cross‐sectional study	Elderly inpatients with type 2 diabetes (≥ 60 years)	A single‐center tertiary hospital	FRAIL	37.1%
Wu Jiaqi et al. [33]	2024	China	Cross‐sectional study	Elderly inpatients with type 2 diabetes (≥ 60 years)	A dual‐center tertiary hospital	FP	29.1%
Wu Liangwen [32]	2023	China	Cross‐sectional study	Elderly inpatients with type 2 diabetes (≥ 60 years)	A single‐center tertiary hospital	EFS	44.6%
Xiao Ruifeng et al. [35]	2025	China	Retrospective study	Elderly patients with diabetes from CHARLS database (≥ 60 years)	CHARLS	FP	10.2%

Abbreviations: CHARLS, China Health and Retirement Longitudinal Study database; EFS, Edmonton Frailty Scale; FP, Fried frailty phenotype; FRAIL, FRAIL Scale; TFI, Tilburg Frailty Index.

### 3.3. Model Development and Predictors

The sample size of the included studies ranged from 152 to 1436 participants, and the number of outcome events (frailty cases) ranged from 64 to 229. Various statistical and machine‐learning approaches were used for model development, including logistic regression, nomograms, classification and regression trees (CART), artificial neural networks, random forests, support vector machines (SVM), k‐nearest neighbors (KNN), extreme gradient boosting (XGBoost) model, categorical boosting (XGBoost) model, and light gradient boosting machine (LightGBM) model. Logistic regression was the most frequently used modeling method [19–25, 27–29, 31, 32, 34, 35, 37]. Events per variable (EPV) was calculated using the dataset employed for model development and was defined as the number of outcome events divided by the number of predictor parameters included in the final model. Eleven studies met the EPV criterion (EPV ≥ 20) [19, 21, 24, 26, 27, 29, 31–34, 37], whereas eight studies did not (EPV < 20) [20, 22, 23, 25, 28, 30, 35, 36]. Only nine studies reported the handling of missing data. Six studies [19, 20, 23, 27, 29, 35] excluded participants with missing data, whereas three studies [25, 33, 36] used multiple imputation. The most frequently reported predictors included age (*n* = 10), depression (*n* = 9), activities of daily living (ADL*n* = 7), nutritional status (*n* = 6), duration of diabetes (*n* = 6), physical exercise or physical activity (*n* = 6), polypharmacy (*n* = 5), glycated hemoglobin (HbA1c;*n* = 5), cognitive function or cognitive impairment (*n* = 5), and marital status–related factors (*n* = 5). Detailed characteristics of the included models are presented in Table 2.

**Table 2:** Overview of the information of the included prediction models (*n* = 19).

Author	Modeling methods	Count of final predictors	Final predictors	Sample size	Outcome cases	Missing data		EPV
						Number	Method	
Bu F et al. [19]	LR	7	Marital status, ADL, waist circumference, cognitive function, grip strength, social activity, and depression	1005/431	145	No report	Excluded	20.71
Yin YuanYuan [21]	LR (nomogram) + CART	8/3	LR model (eight predictors): Cognitive function, polypharmacy, depression, CCI score, per capita monthly family income, self‐management behavior, gender, and HbA1c	379	194	6	No report	24.25
			CART model (three predictors): cognitive function, ADL, and depression					
Dang Xue [22]	LR	5	Age, social support, history of falls, hypoglycemia, and polypharmacy	252/108	88/27	No report	No report	17.6
Liu Xi et al. [23]	LR	6	Age, regular exercise, diabetes duration, HbA1c level, depression, and nutritional status	462/197	131/54	No report	Exclusion of individuals with excessive missing data (exceeding 20%).	16.4
Tang Qingfeng et al. [24]	LR	6	Age, duration of diabetes, HbA1c, number of hypoglycemic episodes, diabetic foot, and marital status	351/215	132/‐	No report	No report	22
Xi Mengxing [25]	LR	6	Age, duration of diabetes, HbA1c, ADL, movement dysfunction, hospitalization due to diabetes exacerbation in the past year, and presence of ≥ 4 comorbid chronic diseases	237/101	90/40	9	Multiple imputation	15
Li Teng [26]	LR, K‐NNM, RF, EGBM	6	Age, duration of diabetes, regular exercise (yes/no), depression status, nutritional status, and comorbidity status	400	177	0	Excluded	29.5
Du Jin et al. [20]	LR	7	Age, living alone (yes/no), regular exercise (yes/no), depression (yes/no), nutritional status, sleep condition, and HbA1c level	527	64	7	Subjects with missing data were eliminated	9.13
Wang Bingjie et al. [27]	LR, ANN	8	Regular exercise, comorbidities, polypharmacy, foot ulcer, fasting blood glucose, glomerular filtration rate (GFR), nutritional status, and self‐management level	491	216	No report	Exclusion of > 10% missing data	27
Wang Zhuo et al. [28]	LR	4	Gender (female), comorbid stroke, depressive symptoms, and high risk of falling	106/46	33/14	No report	No report	8.25
Dong Xuting et al. [29]	LR	7	Marital status, living status, exercise habits, presence of other diabetic complications, duration of diabetes, coping strategies, and loneliness score	485	211	15	No report	21
Zheng Xuemei et al. [30]	RF, SVM, K‐NNM	7	Multimorbidity, polypharmacy, ADL, self‐management ability, nutritional status, 25‐hydroxyvitamin D_3_, and diabetes durationL	380	112	No report	No report	16
Chen Youmei [31]	LR	5	Night sleep duration, physical exercise, chronic pain, depression, and mild self‐care dependence	317	118	3	No report	23.6
Deng Shunzhi et al. [36]	XGBoost, SVM, LR	7	Cognitive impairment, CCI, chronic pain, physical exercise, sarcopenia, nutritional status, and diabetic nephropathy	227	99	No report	Random forest imputation (for missing rate < 10%)	14.1
Ma Bingying [37]	LR	5	Age, physical activity, albumin, ADL, and depression	370/125	135/21	No report	No report	27
Wang Weijia [34]	LR	5	Age, polypharmacy, HDL, nutritional status, and TCM syndrome type	345	128	5	No report	25.6
Wu Jiaqi et al. [33]	CatBoost, LightGBM, RF, SVM, LR	7	Depression, age, GNRI, hemoglobin, self‐management, sleep, and HbA1c	509	148	No report	Multiple imputation (missing rate of less than 30%)	21.1
Wu Liangwen [32]	LR	6	Widowhood, depression, glucosuria, ADL, CCI, and fasting blood glucose	514	229	No report	No report	26.5
Xiao Ruifeng et al. [35]	LR	7	ADL, cognition, grip strength, waist circumference, absence of a spouse, chronic lung disease, and low social activity	1107	113	329	Exclusion of missing data	16.1

Abbreviations: 25‐OHD_3_, 25‐hydroxyvitamin D_3_; ADL, activities of daily living; ALB, albumin; ANN, artificial neural network; CART, Classification and Regression Tree; CatBoost, categorical boosting model; CCI, Charlson comorbidity index; EGBM, extreme gradient boosting model; GNRI, The Geriatric Nutritional Risk Index; HbA1c, glycated hemoglobin A1c; HDL, High‐density lipoprotein; K‐NNM, K‐nearest neighbors models; LightGBM, light gradient boosting machine model; LR, logistic regression; RF, random forest; SVM, support vector machine; XGBoost, extreme gradient boosting model.

### 3.4. Predictive Performance of the Model

AUC is commonly used as an indicator of a model′s discriminative ability. The AUC values of the included models ranged from 0.703 to 0.975. Among them, the random forest model developed by Li Teng [26] demonstrated the highest discriminative performance, outperforming both traditional logistic regression and other machine‐learning models, with an AUC of 0.975. All included studies reported AUC values; however, three studies [26, 30, 36] did not provide corresponding 95% CIs. Instead, sensitivity, specificity, and accuracy were additionally reported as measures of model performance. Nine studies [21, 22, 25, 26, 28, 29, 31, 34, 37] assessed calibration using both the Hosmer–Lemeshow goodness‐of‐fit test and calibration plots. One study [32] evaluated calibration using the Hosmer–Lemeshow goodness‐of‐fit test, calibration plots, and the Brier score. Two studies [19, 23] assessed calibration solely using the Hosmer–Lemeshow goodness‐of‐fit test, whereas three studies [20, 35, 36] reported calibration using calibration plots only. The remaining three studies [27, 30, 33] did not report any calibration assessment methods. Overall, discrimination was consistently reported across studies, whereas calibration assessment and reporting showed considerable variability.

Regarding model validation, internal validation was the most commonly applied approach. A total of 18 studies [19–21, 23–37] performed internal validation, including bootstrap resampling, cross‐validation, and random split‐sample validation. Only three studies [26, 32, 37] conducted both internal and external validation. In contrast, one study [22] performed temporal validation as an external validation method without conducting any internal validation. Prediction models were presented using various approaches, including risk score formulas, Python‐based models, nomograms, SHAP (Shapley Additive Explanations) plots, and decision trees. Details of model performance are provided in Table 3.

**Table 3:** Performance and predictive factors of the frailty risk prediction model for diabetic patients (*n* = 19).

Included literature	Discrimination			Mold validation method		Model presentation
	AUC (95% CI) in the development cohorts	AUC (95% CI) in the validation cohorts	Calibration method	Internal validation	External validation	
Bu F et al. [19]	LR modeling set: 0.912	LR modeling set: 0.887–0.937	Hosmer–Lemeshow goodness‐of‐fit test (*p* = 0.824)	Random split‐sample validation	None	Risk nomogram
	LR validation set: 0.881	LR validation set: 0.829–0.934				
Yin YuanYuan [21]	LR (nomogram) modeling set: 0.865	LR (nomogram) modeling set: 0.860–0.940	Hosmer–Lemeshow goodness‐of‐fit test (*p* = 0.231), calibration plot	Bootstrap resampling	None	Risk nomogram, decision tree
	LR (nomogram) validation set: 0.872	LR (nomogram) validation set: 0.870–0.873				
	CART modeling set: 0.783	CART modeling set: 0.743–0.823				
	CART validation set: 0.813	CART Validation set: 0.810–0.815				
Dang Xue [22]	LR modeling set: 0.900	LR modeling set: 0.860–0.940	Hosmer–Lemeshow goodness‐of‐fit test (*p* = 0.123), calibration plot	None	Period verification	Risk nomogram
	LR validation set: 0.890	LR validation set: 0.830–0.950				
Liu Xi et al. [23]	LR validation set: 0.758	LR validation set: 0.674–0.842	Hosmer–Lemeshow goodness‐of‐fit test (*p* > 0.05)	Random split‐sample validation	None	Risk scoring formula
Tang Qingfeng et al. [24]	LR modeling set: 0.790	LR modeling set: 0.742–0.838	Hosmer–Lemeshow goodness‐of‐fit test (*p* = 0.962), calibration plot	Random split‐sample validation	None	Risk nomogram
	LR validation set: 0.703	LR validation set: 0.628–0.778				
Xi Mengxing [25]	LR modeling set: 0.851	LR modeling set: 0.799–0.902	Hosmer–Lemeshow goodness‐of‐fit test (*p* = 0.099), calibration plot	Random split‐sample validation	None	Risk scoring formula, risk nomogram
	LR validation set: 0.824	LR validation set: 0.732–0.906				
Li Teng [26]	LR modeling set: 0.964	No report	Hosmer–Lemeshow goodness‐of‐fit test, calibration plot	Cross‐validation	Verified by the NHANES database	Risk scoring formula, R language model
	RF modeling set: 0.975					
	RF validation set: 0.810					
	K‐NNM method modeling set: 0.953					
	EGBM modeling set: 0.959					
Du Jin et al. [20]	LR modeling set: 0.768 LR	LR modeling set: 0.714–0.822	Calibration plot	Bootstrap resampling	None	Risk nomogram
	Validation set: 0.732					
Wang Bingjie et al. [27]	LR modeling set: 0.973	LR modeling set: 0.950–0.987	No report	Random split‐sample validation	None	Risk nomogram
	LR validation set: 0.964	LR validation set: 0.919–0.988				
	ANN modeling set: 0.742	ANN modeling set: 0.693–0.788				
	ANN validation set: 0.732	ANN validation set: 0.653–0.802				
Wang Zhuo et al. [28]	LR modeling set: 0.840	LR modeling set: 0.765–0.915	Hosmer–Lemeshow goodness‐of‐fit test (*p* = 0.345), calibration plot	Random split‐sample validation	None	Risk nomogram
	LR validation set: 0.933	LR validation set: 0.864–1.000				
Dong Xuting et al. [29]	LR modeling set: 0.763	LR modeling set: 0.711–0.814	Hosmer–Lemeshow goodness‐of‐fit test (*p* = 0.537), calibration plot	Bootstrap resampling	None	Risk nomogram
	LR validation set: 0.766					
Zheng Xuemei et al. [30]	RF modeling set: 0.85	No report	No report	Cross‐validation	None	Python‐based model
	K‐NNM modeling set: 0.79					
	SVM modeling set: 0.83					
Chen Youmei [31]	LR modeling set: 0.969	LR modeling set: 0.950–0.987	Hosmer–Lemeshow goodness‐of‐fit test (*p* = 0.496), calibration plot	Bootstrap resampling	None	Risk scoring formula, risk nomogram
Deng Shunzhi et al. [36]	XGBoost modeling set: 0.92	No report	Calibration plot	Bootstrap resampling	None	SHAP plot
Ma Bingying [37]	LR modeling set: 0.858	LR modeling set: 0.813–0.902	Hosmer–Lemeshow goodness‐of‐fit test (*p* = 0.515), calibration plot	Bootstrap resampling	Time‐based validation	Risk nomogram
	LR validation set: 0.822	LR validation set: 0.770–0.874				
Wang Weijia [34]	LR modeling set: 0.933	LR modeling set: 0.905–0.961	Hosmer–Lemeshow goodness‐of‐fit test(*p* = 0.533), calibration plot	Bootstrap resampling	Time‐based validation	Risk nomogram
	LR validation set: 0.933	LR validation set: 0.899–0.958				
Wu Jiaqi et al. [33]	CatBoost validation set: 0.755	CatBoost validation set: 0.752 to 0.757	No report	Cross‐validation	No report	SHAP plot
	LR validation set: 0.741	LR validation set: 0.738–0.742				
	SVM validation set: 0.740	SVM validation set: 0.730–0.749				
	LightGBM validation set: 0.748	LightGBM validation set: 0.742–0.754				
	RF validation set: 0.751	RF validation set: 0.748–0.754				
Wu Liangwen [32]	LR modeling set: 0.814	LR modeling set: 0.772–0.857	Hosmer–Lemeshow goodness‐of‐fit test (*p* = 0.192), calibration plot, Brier score 0.181	Bootstrap resampling	Time‐based validation	Risk nomogram
	LR validation set: 0.879	LR validation set: 0.821–0.927				
Xiao Ruifeng et al. [35]	LR modeling set: 0.879	LR modeling set: 0.838–0.920	Calibration plot	Random split‐sample validation	No report	Risk nomogram

### 3.5. Meta‐Analysis Results

#### 3.5.1. Meta‐Analysis of Model AUC

**Figure 3:**
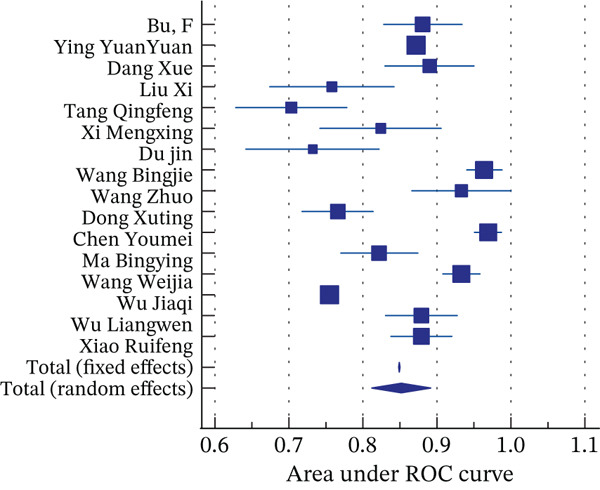
Forest plot of the pooled AUC of frailty prediction models in patients with diabetes.

**Figure 4:**
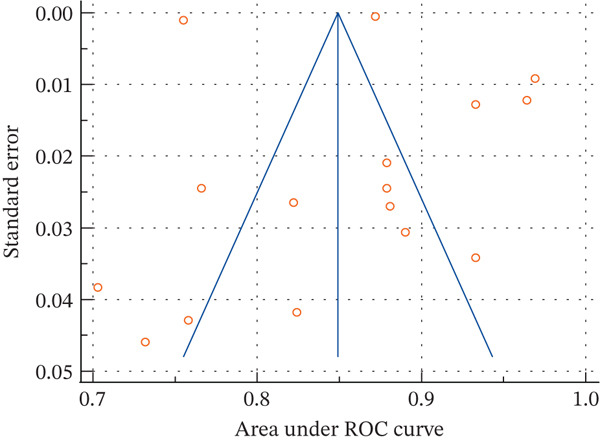
Funnel plot assessing publication bias among the included studies.

Due to the lack of 95% CIs for model performance in three studies [26, 30, 36], these studies were excluded from the quantitative synthesis requiring standard error estimation. A meta‐analysis of the remaining 16 studies [19–25, 27–29, 31–35, 37] was performed. Heterogeneity analysis indicated substantial heterogeneity among the included models (*p* < 0.0001, *I* ^2^ = 99.86*%*), and a random‐effects model was therefore applied. The pooled AUC was 0.852 (95% CI: 0.812–0.892), as shown in Figure 3. Although significant heterogeneity was observed, sensitivity analyses demonstrated that the pooled results were stable and robust. Egger′s regression test indicated no significant publication bias (*p* > 0.05); however, the reliability of this result may be limited due to the high heterogeneity. Visual inspection of the funnel plot did not reveal obvious asymmetry (Figure 4). Subgroup analyses were not conducted due to the limited number of studies within subgroups and substantial variability in assessment tools, predictor selection strategies, model development methods, and validation approaches.

#### 3.5.2. Meta‐Analysis of Predictors

A meta‐analysis was performed to evaluate the association between common predictors and frailty in patients with diabetes, including age, depression, ADL, HbA1c, polypharmacy, duration of diabetes, physical exercise, cognitive function, sleep status, comorbidities, gender, marital status, nutritional status, self‐management ability, history of hypoglycemia, fasting blood glucose, grip strength, waist circumference, diabetic complications, and serum albumin. The results indicated that age, ADL, depression, polypharmacy, longer duration of diabetes, higher HbA1c levels, comorbidities, lower self‐management ability, history of hypoglycemia, elevated fasting blood glucose, reduced grip strength, increased waist circumference, female sex, diabetic complications, impaired cognitive function, and lower serum albumin were significantly associated with frailty in patients with diabetes (*p* < 0.05). Significant heterogeneity was observed across studies; therefore, a random‐effects model was applied. Sensitivity analyses, conducted by sequentially excluding individual studies, showed that the pooled estimates remained stable, indicating the robustness of the results.

### 3.6. Risk of Bias and Applicability Assessment

**Figure 5:**
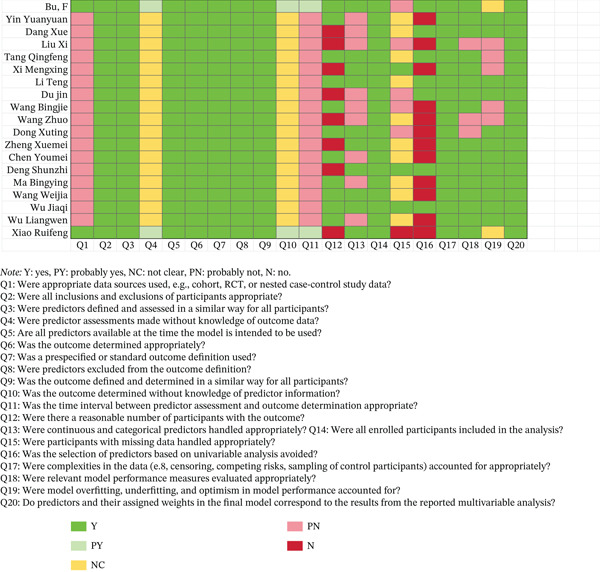
Risk of bias assessment by using PROBAST across 20 signaling questions. Note: Y, yes; PY, probably yes; NC, not clear; PN, probably not; N, no. Q1: Were appropriate data sources used, for example, cohort, RCT, or nested case–control study data? Q2: Were all inclusions and exclusions of participants appropriate? Q3: Were predictors defined and assessed in a similar way for all participants? Q4: Were predictor assessments made without knowledge of outcome data? Q5: Are all predictors available at the time the model is intended to be used? Q6: Was the outcome determined appropriately? Q7: Was a prespecified or standard outcome definition used? Q8: Were predictors excluded from the outcome definition? Q9: Was the outcome defined and determined in a similar way for all participants? Q10: Was the outcome determined without knowledge of predictor information? Q11: Was the time interval between predictor assessment and outcome determination appropriate? Q12: Were there a reasonable number of participants with the outcome? Q13: Were continuous and categorical predictors handled appropriately? Q14: Were all enrolled participants included in the analysis? Q15: Were participants with missing data handled appropriately? Q16: Was the selection of predictors based on univariable analysis avoided? Q17: Were complexities in the data (e.g, censoring, competing risks, sampling of control participants) accounted for appropriately? Q18: Were relevant model performance measures evaluated appropriately? Q19: Were model overfitting, underfitting, and optimism in model performance accounted for? Q20: Do predictors and their assigned weights in the final model correspond to the results from the reported multivariable analysis?

The results of the risk of bias and applicability assessment using the PROBAST tool are presented in Table 4 and Figure 5.

**Table 4:** Evaluation results of the bias risk and applicability of the included studies (*n* = 19).

Included literature	Risk of bias				Applicability			Assessment	
	Study subjects	Predictor	Result	Data analysis	Study subjects	Predictor	Result	Risk of bias	Applicability
Bu F et al. [19]	Low	Low	Low	High	Low	Low	Low	High	Low
Yin YuanYuan [21]	High	High	High	High	Low	Low	Low	High	Low
Dang Xue [22]	High	High	High	High	Low	Low	Low	High	Low
Liu Xi et al. [23]	High	High	High	High	Low	Low	Low	High	Low
Tang Qingfeng et al. [24]	High	High	High	High	Low	Low	Low	High	Low
Xi Mengxing [25]	High	High	High	High	Low	Low	Low	High	Low
Li Teng [26]	High	High	High	High	Low	Low	Low	High	Low
Du Jin et al. [20]	High	High	High	High	Low	Low	Low	High	Low
Wang Bingjie et al. [27]	High	High	High	High	Low	Low	Low	High	Low
Wang Zhuo et al. [28]	High	High	High	High	Low	Low	Low	High	Low
Dong Xuting et al. [29]	High	High	High	High	Low	Low	Low	High	Low
Zheng Xuemei et al. [30]	High	High	High	High	Low	Low	Low	High	Low
Chen Youmei [31]	High	High	High	High	Low	Low	Low	High	Low
Deng Shunzhi et al. [36]	High	High	High	High	Low	Low	Low	High	Low
Ma Bingying [37]	High	High	High	High	Low	Low	Low	High	Low
Wang Weijia [34]	High	High	High	High	Low	Low	Low	High	Low
Wu Jiaqi et al. [33]	High	High	High	Low	Low	Low	Low	High	Low
Wu Liangwen [32]	High	High	High	High	Low	Low	Low	High	Low
Xiao Ruifeng et al. [35]	Low	Low	Low	High	Low	Low	Low	High	Low

#### 3.6.1. Participants Domain

In the participants domain, 17 studies [20–34, 36, 37] were rated as having a high risk of bias. All of these studies employed a cross‐sectional design. As a result, limitations in sampling strategies may have led to potential selection bias and reduced representativeness of the target population. In addition, the cross‐sectional nature of these studies implies that predictor and outcome information were collected within the same time frame, which may introduce additional bias in the study population domain. In contrast, two studies based on the CHARLS database [19, 35] were rated as having a low risk of bias, as they were derived from a large‐scale, population‐based cohort dataset with relatively more representative sampling of the target population.

#### 3.6.2. Predictors Domain

In the predictors domain, 17 studies [20–34, 36, 37] were judged to have a high risk of bias due to a lack of reporting on whether predictor assessment was performed blinded to outcome information. Although standardized assessment tools were applied in most studies, the absence of blinding may have introduced potential information bias. In addition, in many cross‐sectional studies, predictors and outcomes were assessed within the same time frame, often by the same investigators or using the same data collection process, which may have further increased the risk of bias in predictor measurement. In contrast, two studies based on the CHARLS database [19, 35] were judged to have a low risk of bias. These studies derived predictor information from baseline assessments that preceded outcome ascertainment, thereby reducing the likelihood of information bias related to outcome awareness during predictor measurement.

#### 3.6.3. Outcome Domain

In the outcome domain, 17 studies [20–34, 36, 37] were rated as having a high risk of bias, whereas two studies [19, 35] were rated as having a low risk of bias. The 17 studies with high risk of bias did not report whether outcome assessment was performed blinded to predictor information. Although standardized frailty assessment tools were used in most studies, the lack of reporting on blinding may have introduced potential detection bias in outcome measurement. In addition, these studies were based on cross‐sectional data, in which predictor and outcome information were collected at the same time point. This may limit the ability to clearly distinguish the temporal relationship between predictors and outcomes. In contrast, the two studies based on the CHARLS database [19, 35] were rated as having a low risk of bias, as outcome and predictor information were derived from a cohort dataset with a clearer temporal structure between baseline assessment and outcome evaluation.

#### 3.6.4. Analysis Domain

Eighteen studies [19–33, 35–37] (94.7%) were rated as having a high risk of bias in the analysis domain, whereas only one study [34] (5.3%) was rated as having a low risk of bias. This was the most prominent methodological limitation identified across the included studies. Eleven studies met the recommended criterion of at least 20 events per predictor variable (EPV ≥ 20) [19, 21, 24, 26, 27, 29, 31–34, 37]. In contrast, eight studies had an EPV below 20, which may have increased the risk of model overfitting [20, 22, 23, 25, 28, 30, 35, 36]. The lowest EPV was 8.25 [28]. In addition, nine studies categorized continuous predictors [20–23, 27, 28, 31, 32, 37], which may have led to a loss of information and reduced statistical power.

Only three studies used appropriate methods for handling missing data. Two studies employed multiple imputation [25, 33], and one study used random forest imputation [36]. Six studies excluded participants with missing data through complete‐case analysis [19, 20, 23, 27, 29, 35], which may have introduced selection bias. The remaining 10 studies did not report any information regarding the handling of missing data [21, 22, 24, 26, 28, 30–32, 34, 37].

Regarding predictor selection, 12 studies (63.2%) used univariable analysis to screen candidate predictors before multivariable logistic regression modeling [21, 23, 25, 27–32, 34, 35, 37]. This approach may exclude predictors that are nonsignificant in univariable analyses but become important after adjustment for other variables in multivariable models. Only seven studies used appropriate multivariable predictor selection methods, including LASSO regression [19, 20, 22, 24, 33, 36] or a combination of multiple variable‐selection methods [26].

Although all 19 studies reported model discrimination using the AUC, only 14 studies adequately assessed model calibration through calibration plots [19–22, 24–26, 28, 29, 31, 32, 34, 35, 37]. Furthermore, only nine studies evaluated clinical utility using decision curve analysis (DCA) [19, 20, 22, 24–26, 32, 34, 35]. Appropriate internal validation methods to address potential overfitting and optimism bias in model performance were employed in 11 studies, including bootstrap resampling [20, 21, 26, 29, 31, 32, 34, 36, 37] and cross‐validation [30, 33]. In contrast, seven studies relied solely on random split‐sample validation [23–25, 27, 28, 35], a method generally considered inadequate for evaluating optimism in model performance estimates. Only four studies conducted external validation, with one study [26] using an independent external dataset (NHANES database) and three studies [22, 32, 37] performing temporal validation across different time periods. The remaining 15 studies performed internal validation only, which may limit the generalizability of their prediction models.

#### 3.6.5. Applicability

All 19 studies were judged to have low concerns regarding applicability, indicating good alignment with the review question in terms of participants, predictors, and outcomes. In the participants domain, all studies focused on patients with diabetes, which was consistent with the target population of this review. However, several studies were conducted in specific subgroups of patients with diabetic complications, such as diabetic foot [27] and diabetic retinopathy [29], which should be taken into account when interpreting the generalizability of their findings.

In the predictors domain, all studies included clinically accessible predictors, such as age, depression, ADL, and nutritional status. Nevertheless, variations existed in the definitions and measurement methods of some predictors across studies. For example, different instruments were used to assess nutritional status.

In the outcome domain, all studies employed standardized frailty assessment tools. However, differences in the domains covered and cutoff values used by these instruments may have affected the direct comparability of findings across studies. Overall, the included studies were considered highly relevant to the review question, and concerns regarding applicability were judged to be low.

## 4. Discussion

To our knowledge, this is the first systematic review evaluating frailty prediction models in patients with diabetes. Nineteen studies were included. The identified models demonstrated good to excellent discriminative performance (AUC: 0.703–0.975), and the most frequently reported predictors were summarized. Despite their promising performance, all studies were judged to be at high risk of bias, primarily because of cross‐sectional designs and methodological limitations in the analysis domain. Further external validation, model refinement, and impact assessment studies are required before widespread clinical implementation.

Across the included studies, the most frequently reported predictors of frailty in patients with diabetes could be grouped into nonmodifiable and modifiable factors. Nonmodifiable factors included age and duration of diabetes. Modifiable factors included depressive symptoms, impaired ADL, polypharmacy, poor glycemic control, cognitive impairment, comorbidities, self‐management ability, and fasting blood glucose. The identification of several potentially modifiable predictors suggests opportunities for targeted interventions to reduce frailty risk and improve health outcomes in this population.

Previous evidence has consistently identified advancing age as an important risk factor for frailty [38, 39]. Given the heterogeneity in frailty risk across different age groups and stages of disease progression, future studies should consider developing and validating prediction models stratified by age and duration of diabetes. Such an approach may improve model performance, enhance clinical applicability, and facilitate more individualized risk assessment.

In contrast, depressive symptoms, polypharmacy, cognitive impairment, reduced ADL, and poor glycemic control are potentially modifiable factors and therefore represent important targets for intervention [39–42]. Early identification and management of these factors may help prevent or delay frailty progression. These findings support a personalized approach to frailty prevention in patients with diabetes, emphasizing routine assessment of functional status, mental health, medication burden, and glycemic control. Individualized interventions targeting these domains may improve patient outcomes and reduce frailty risk.

The included studies used a variety of frailty assessment instruments, including the FP, FRAIL Scale, TFI, and EFS [19–37]. Although these tools differ in their conceptual frameworks and measurement domains, all are validated instruments for identifying frailty and were therefore considered eligible for inclusion. This heterogeneity in assessment instruments may contribute to variations in reported frailty prevalence and differences in model performance across studies. In addition, some studies used modified versions of these instruments, which may have increased the risk of outcome misclassification [19, 35]. Greater standardization of frailty assessment methods would facilitate cross‐study comparisons and support the development of more robust and generalizable prediction models.

Although substantial heterogeneity was observed in the pooled analysis, sensitivity analyses demonstrated that the overall findings were stable. No significant publication bias was detected by Egger′s regression test. The observed heterogeneity is likely attributable to differences in assessment instruments, predictor selection strategies, model development methods, and validation procedures across studies. Therefore, the pooled AUC should be interpreted with caution, as it reflects the average discriminative performance across heterogeneous prediction models rather than the performance of a single model intended for clinical use. Subgroup analyses were considered but were not performed because the number of studies within individual categories remained limited after stratification, which could have resulted in unstable and potentially misleading estimates. Future studies with a larger evidence base may allow more informative subgroup analyses to further investigate potential sources of heterogeneity.

Despite generally good predictive performance, all included studies were judged to be at high risk of bias. Major methodological concerns included the use of cross‐sectional designs, inadequate handling of missing data, inappropriate predictor selection methods, categorization of continuous variables, and limited external validation. These limitations may have resulted in overly optimistic estimates of model performance and restricted model generalizability. Future studies should prioritize prospective cohort designs, rigorous handling of missing data, adequate sample sizes, and both internal and external validation in accordance with established guidance such as PROBAST [18] and TRIPOD [43].

Five studies applied machine‐learning algorithms [26, 27, 30, 33, 36]. Compared with conventional logistic regression models, machine‐learning approaches may better capture complex nonlinear relationships and interactions among predictors, potentially improving predictive performance. Several studies further incorporated SHAP analyses to improve model interpretability [33, 36]. Nevertheless, machine‐learning models remain vulnerable to overfitting and require rigorous validation before clinical implementation [44]. Future research should evaluate not only predictive performance but also the interpretability, stability, and clinical utility of these approaches.

The findings of this review have important implications for clinical practice and future research. The identified modifiable predictors, including depression, polypharmacy, impaired activities of daily living, poor glycemic control, and cognitive decline, may provide practical targets for individualized frailty prevention and management strategies in patients with diabetes. Early identification of high‐risk individuals may facilitate timely intervention and support more proactive, person‐centered care.

Future research should focus on developing and validating prediction models using prospective multicenter cohorts and more diverse populations. Greater attention should be paid to external validation, model updating, and implementation studies to determine whether frailty prediction models can improve clinical decision‐making and patient outcomes. In addition, advances in machine learning and explainable artificial intelligence may offer opportunities to enhance both prediction accuracy and model interpretability.

## 5. Limitation

This review has several limitations. All included prediction models were developed and validated in Chinese populations, which may limit the generalizability of the findings to other ethnic, cultural, and healthcare settings. Although these studies provide important evidence regarding frailty risk among patients with diabetes in China, the geographical homogeneity of the evidence base may introduce systematic bias.

Patients with diabetes in China may differ from those in other countries with respect to demographic characteristics, genetic background, dietary patterns, socioeconomic status, family support systems, and healthcare delivery models. These differences may influence both the distribution of predictors and their associations with frailty. Consequently, the predictive performance and effect estimates reported in the included studies may not be directly transferable to other populations. Furthermore, even when model discrimination remains acceptable, calibration performance may deteriorate when models are applied to populations that differ substantially from the development cohort, potentially resulting in inaccurate risk estimates. Therefore, large‐scale prospective external validation studies in diverse populations, particularly in East Asian and Western countries, are needed to evaluate the transportability and clinical applicability of existing frailty prediction models.

All included studies were assessed as having a high risk of bias according to the PROBAST tool. Although this finding highlights important methodological limitations, it also reflects the current stage of development of frailty prediction research in patients with diabetes. Most studies adopted cross‐sectional or retrospective designs, and eight were master′s theses [21, 22, 25, 26, 31, 32, 34, 37], which may affect the strength and generalizability of the evidence. In addition, reporting and handling of missing data were often inadequate across the included studies. Inappropriate management of missing data may introduce bias, reduce statistical efficiency, and compromise the validity of prediction models [45]. Nevertheless, by transparently evaluating and reporting these limitations using a standardized assessment tool, this review provides a methodological benchmark for the design and reporting of future prediction model studies.

The limited number of externally validated models may further restrict their clinical applicability. Only four [22, 26, 32, 37] of the 19 [19–37] included studies performed external validation, making it difficult to determine which model is most appropriate for clinical use or to compare model performance across different populations and settings. External validation is a critical step in prediction model research because it provides evidence regarding model reproducibility, transportability, and performance in independent populations [46, 47]. Therefore, additional external validation studies are warranted to strengthen the evidence base and support broader clinical implementation.

To advance the field, future studies should prioritize prospective, multicenter cohort designs with larger and more heterogeneous populations. The integration of longitudinal data may facilitate a better understanding of the dynamic trajectory of frailty over the course of diabetes. In addition, rigorous external validation across diverse populations and healthcare settings is essential to improve model transportability, generalizability, and clinical utility.

## 6. Conclusion

Frailty prediction models in patients with diabetes demonstrated good overall discriminative performance and identified several clinically relevant predictors associated with frailty risk. However, the methodological quality of existing models remains suboptimal, with all included studies being judged at high risk of bias. Future model development and validation studies should adopt more rigorous methodological standards to improve robustness, generalizability, and clinical applicability. With continued methodological refinement, frailty prediction models may become valuable tools for risk stratification and early intervention in patients with diabetes.

## Author Contributions

Qing Chen and Meiling Yang designed the research. Qing Chen, Meiling Yang, and Mengmeng Chen conducted the research. Qing Chen, Meiling Yang, Mengmeng Chen, and Zidan Wang analyzed the data. Qing Chen, Meiling Yang, and Mengmeng Chen wrote the manuscript. Joanne Wai Yee Chung and Chuyuan Miao provided supervision and critically revised the manuscript. Qing Chen, Meiling Yang, and Mengmeng Chen contributed equally to this work and share first authorship.

## Funding

No funding was received for this manuscript.

## Disclosure

All authors read and approved the final manuscript.

## Conflicts of Interest

The authors declare no conflicts of interest.

## Data Availability

All the data used in the study are available from the first and corresponding author on reasonable request.
